# Dexmedetomidine reverses MTX-induced neurotoxicity and inflammation in hippocampal HT22 cell lines via NCOA4-mediated ferritinophagy

**DOI:** 10.18632/aging.202626

**Published:** 2021-02-25

**Authors:** Jingli Chen, Juan Wang, Chenxi Li, Huang Ding, Jishi Ye, Zhongyuan Xia

**Affiliations:** 1 Department of Anesthesiology, Renmin Hospital of Wuhan University, Wuhan 430060, Hubei, China; 2 Department of Anesthesiology, The Central Hospital of Wuhan, Tongji Medical College, Huazhong University of Science and Technology, Wuhan 430060, China; 3 Department of Pain, Renmin Hospital of Wuhan University, Wuhan 430060, Hubei, China; 4 Department of Oral and Maxillofacial Surgery, Laboratory for Tumor Genetics and Regenerative Medicine, The Head and Neurocenter, University Medical Center Hamburg-Eppendorf (UKE), Hamburg 20246, Germany

**Keywords:** dexmedetomidine, NCOA4, ferritinophagy, methotrexate, neuroinflammation

## Abstract

The incidence of chemotherapy-induced cognitive impairment (CICI) has attracted massive attention. Some studies have demonstrated the neuroprotective effects of dexmedetomidine (DEX). Here, alterations in nuclear receptor coactivator 4 (NCOA4)-mediated ferritinophagy were investigated as the possible causes of DEX’s neuroprotection of HT22 cells against methotrexate (MTX)-induced neurotoxicity. We used various concentrations of DEX and NCOA4-siRNA to treat MTX-induced neurotoxicity and inflammation in HT22 cells. The biomarkers of HT22 cells viability, apoptosis and inflammatory were tested. The expression of ferritinophagy markers were detected in the HT22 cells by using western blot and Immunofluorescence. We found that 10 and 50 ng/mL of DEX alleviated MTX-induced hippocampal neuronal inflammatory injuries. Meanwhile, DEX also reversed MTX-induced iron and ROS overproduction. Increasing DEX concentrations caused significant falls in the expression of ferritin heavy chain 1 (FTH1). DEX also increased vital ferritinophagy markers, NCOA4 and LC3II. NCOA4-siRNA transfection annulled the neuroprotective effects of DEX on MTX-induced inflammation in HT22 cells. Additionally, because NCOA4-siRNA disrupted ferritinophagy, DEX’s inhibitory impact on MTX-induced iron and ROS overproduction in HT22 cells was also annihilated. DEX weakened MTX-provoked neurontoxicity in HT22 cells, possibly by improving NCOA4-mediated ferritinophagy. Our discoveries present further mechanisms for understanding the protective effects of DEX against MTX-induced cognitive impairment.

## INTRODUCTION

Chemotherapy-induced cognitive impairment (CICI), also known as “Chemobrain” by the public, can cause a wide variety of cognitive dysfunction symptoms characterized by impaired memory, slow processing multitasking speed, and executive function difficulty. Given that CICI’s incidence is already 17~75% in patients with chemotherapy, the National Cancer Institute has recognized it as one of the most debilitating side effects of cancer treatment [1]. CICI should, therefore, be emphasized and addressed by oncologists and neurologists.

Breast cancer patients and pediatric cancer survivors treated with chemotherapeutic drugs, including methotrexate (MTX), cyclophosphamide, and fluorouracil, are more likely to suffer from CICI, which would affect long-tern prognosis [2, 3]. MTX is a widely used anti-tumor drug, which can reduce the synthesis of tumor cells by inhibiting the reductase of dihydrofolate, and decrease the growth and reproduction of tumor cells [3]. Available studies have focused primarily on the toxic effects of chemotherapy on cerebral white matter and different kinds of glial cells, including microglia, astrocytes, and oligodendrocytes. Other possible mechanisms at play during CICI have, therefore, not been revealed yet.

Nuclear receptor coactivator 4 (NCOA4)-mediated ferritinophagy is a specific autophagic phenomenon and process in which NCOA4 forms complexes—by selectively binding ferritin and autophagosome—that are then degraded in lysosomes to release free iron in cells [4]. NCOA4-mediated ferritinophagy is vital in maintaining the homeostasis of intracellular iron levels and iron availability. Mounting evidence shows that the loss and dysfunction of NCOA4 potentially contribute to the inappropriate accumulation of ferritin and trigger excessive oxidative stress and cytotoxicity in neurodegenerative diseases and cardiovascular diseases [5, 6]. In Parkinson's disease (PD)-associated retinal degeneration, α-synuclein damages ferritinophagy, leading to the accumulation of ferritin and iron overload in the retina [7]. In recent years, ferroptosis has gained added attention from increasing life science researchers, particularly in the field of neuroscience and tumor. Given the critical role of NCOA4-mediated ferritinophagy in modulating iron balance, ferroptosis homeostasis and sensitivity could be regulated by NCOA4. Reportedly, NCOA4-mediated ferritinophagy can also contribute to ferroptosis through the RNA-binding protein ELAVL1/HuR pathway in liver fibrosis [8]. Because of its biological function, ferritinophagy fascinates a growing number of scientists in different fields of medicine. Hence, NCOA4-mediated ferritinophagy’s role in the physiological process of chemotherapy drug-induced neurotoxicity deserves in-depth studies.

Existing studies on the neurotoxicity of MTX have mainly focused on the loss of adaptive myelination in the cerebral cortex, dendritic architecture, and hippocampal neurogenesis [9]. There is presently no study that vividly explores and explains the role of ferritinophagy in MTX-induced neurotoxicity and neuroinflammation.

Dexmedetomidine (DEX) is a common clinical sedative drug with proven neuroprotection and anxiolytic effects [10, 11]. With the number of cancer patients increasing, DEX could be a new alternative in the pharmacotherapy of depressive symptoms for advanced cancer patients. However, whether DEX has an interventional effect on MTX-induced neurotoxicity and neuroinflammation has yet to be proven.

In this study, we sought to test for the protective effect of DEX on the MTX-triggered dysfunction of NCOA4-mediated ferritinophagy, a key step associated with neurotoxicity and inflammation. We also assessed the impact of dexmedetomidine on iron accumulation and mobilization.

## RESULTS

### DEX alleviated MTX-induced cytotoxicity and hippocampal neuronal inflammatory injuries

**Figure 1:**
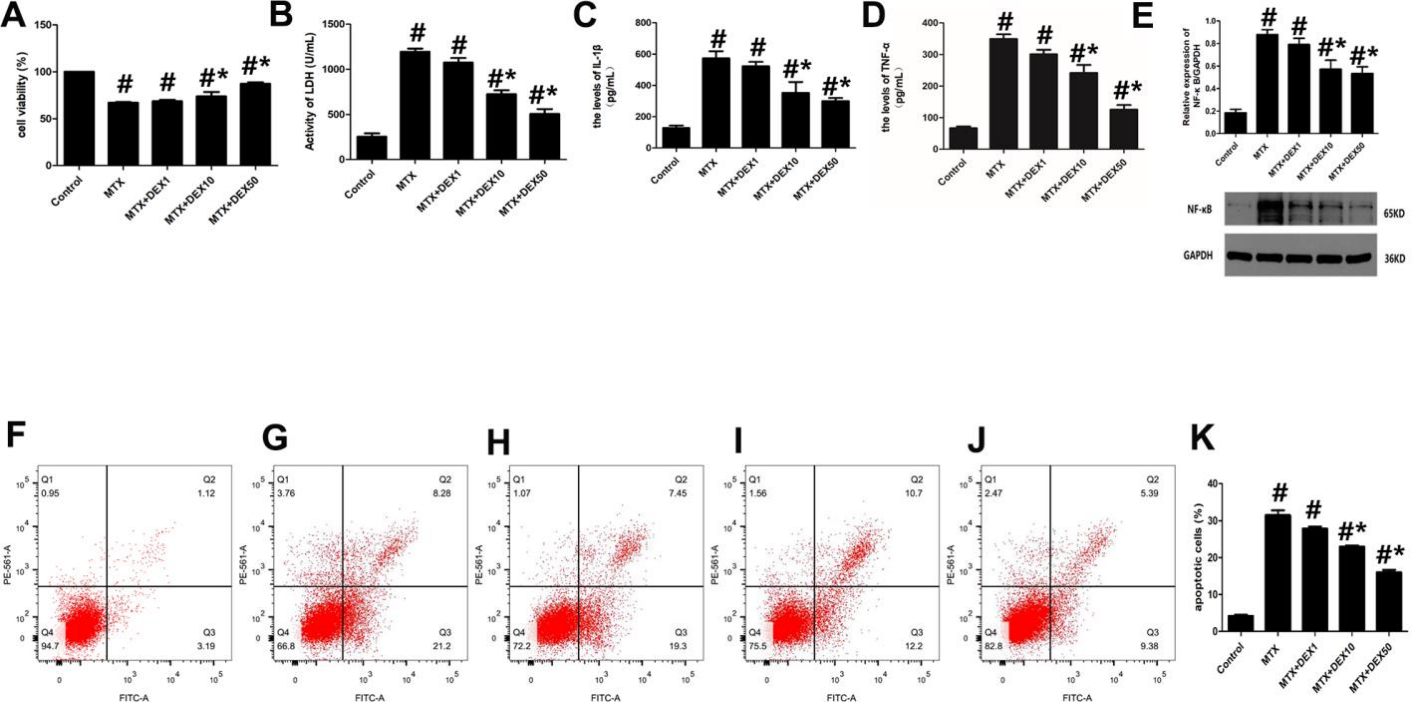
**DEX alleviated cytotoxicity and hippocampal neuronal inflammatory injury induced by MTX**. (**A**) The cell viability of HT22 cells in different groups were measured by CCK-8 assay. (**B**) The activity of LDH in the culture media were measured using LDH assay kit. (**C**) The levels of IL-1β and (**D**) TNF-α in the culture media were measured using ELISA. (**E**) The relative expression of NF-κB in HT22 cells were measured by western-blot. (**F**) The representative results of apoptotic cells in control, MTX (**G**), MTX+1ng/mL DEX (**H**), MTX+10ng/mL DEX (**I**), and MTX+50 ng/mL DEX (**J**) group were tested by flow cytometry. (**K**) Flow cytometry analysis of HT22 cells in different groups. n=3; #p<0.05, vs Control; *p<0.05, vs MTX group.

MTX-induced cytotoxicity in hippocampal neurons was detected by assessing cell viability, LDH release, and apoptotic cells. As shown in Figure 1, MTX caused a significant increase in LDH release in HT22 cells (*P*<0.05). However, 10 and 50 ng/mL of DEX decreased this LDH release dose-dependently (*P*<0.05, Figure 1B). Meanwhile, the number of apoptotic cells was also determined using flow cytometry. Apoptotic cells increased remarkably when HT22 cells were exposed to MTX, compared to the control group (*P*<0.05). However, DEX inhibited this MTX-induced apoptosis in hippocampal neurons (*P*<0.05, Figure1E–1K). Furthermore, the CCK-8 assay showed that 10 and 50 ng/mL of DEX reversed the MTX-induced inhibition of cell viability (*P*<0.05, Figure 1A).

To detect any potential neuronal inflammatory injury caused by MTX, we first examined the impact of MTX on the levels of pro-inflammatory cytokines using ELISA. The results in Figure 1C–1E reveal that MTX significantly up-regulated the levels of TNF-α and IL-1β compared to the control group (*P*<0.05). 10 and 50 ng/mL of DEX suppressed MTX-provoked inflammatory cytokine secretions in HT22 cells (*P*<0.05). Similarly, NF-κB was also enhanced in HT22 cells exposed to MTX compared to the control group (*P*<0.05). However, NF-κB was inhibited in HT22 cells treated with DEX (*P*<0.05). These findings suggest that 10 and 50 ng/mL of DEX can alleviate MTX-induced hippocampal neuronal inflammatory injuries.

### DEX reduced MTX-induced iron overload and ROS overproduction in HT22 cells

**Figure 2:**
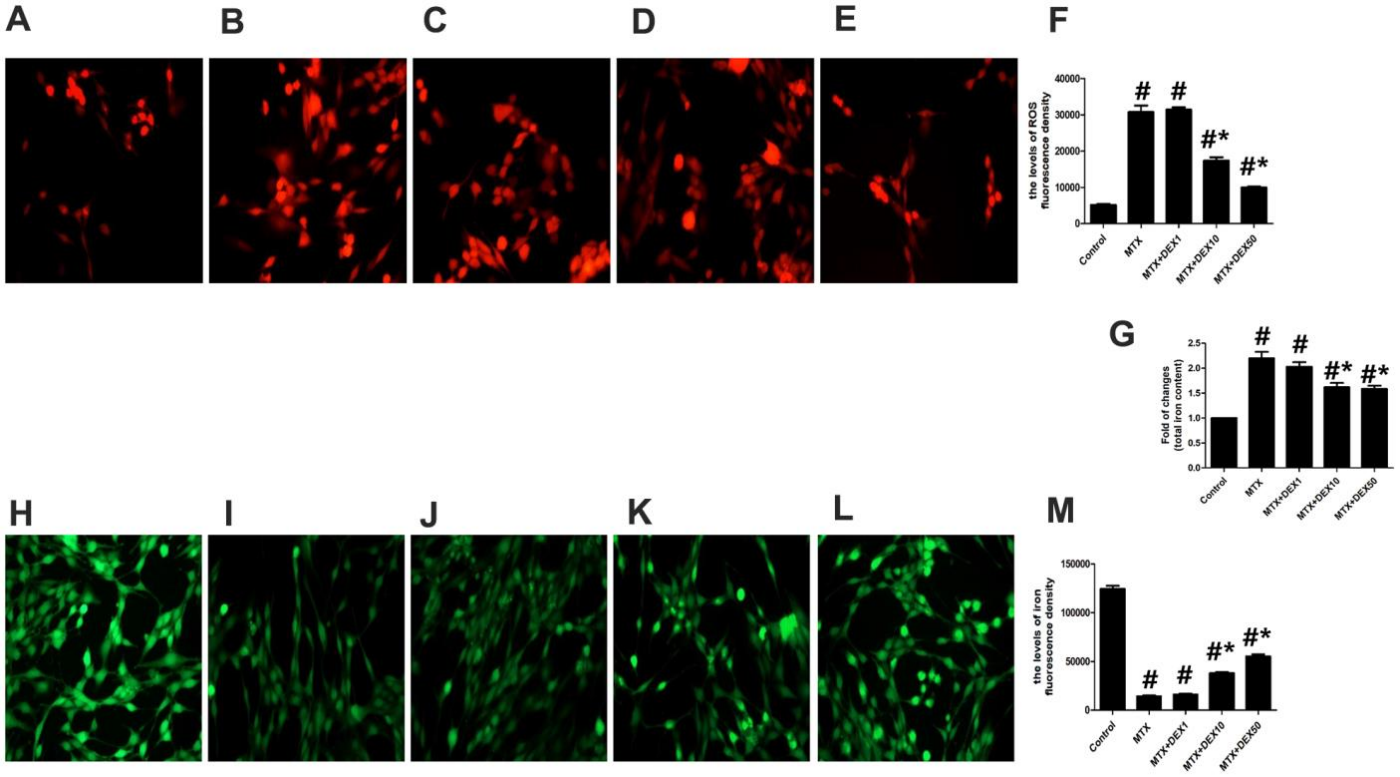
**DEX ameliorated MTX-induced iron overload and ROS overproduction in HT22 cells**. (**A**–**E**) Fluorescence microscope with ROS Probe analysis and representative results of ROS in HT22 cells with different treatments. (**F**) Quantitative results of ROS fluorescence intensity in HT22 cells with different treatments. n=3; #p<0.05, vs Control; *p<0.05, vs MTX group. (**G**) Quantitative results of total iron content in HT22 cells with different treatments. n=3; #p<0.05, vs Control; *p<0.05, vs MTX group. (**H**–**L**) Fluorescence microscope with Ca-AM probe analysis and representative results of iron content in HT22 cells with different treatments. (**M**) Quantitative results of Ca-AM fluorescence intensity in HT22 cells with different treatments. n=3; #p<0.05, vs Control; *p<0.05, vs MTX group.

Cytotoxicity and inflammatory injuries are usually accompanied by excessive oxidative stress and iron overload. To detect further protective effects of DEX, we measured the content of total iron, labile iron pool, and ROS production in HT22 cells. As shown in Figure 2A–2F, MTX significantly increased ROS production in HT22 cells compared to the control group (*P*<0.05), which was consistent with findings from cytotoxicity tests. However, 10 and 50 ng/mL of DEX significantly reduced MTX-induced ROS production in HT22 cells (*P*<0.05). MTX also notably augmented the levels of the labile iron pool and total iron content in HT22 cells (*P*<0.05, Figure 2G–2M), but 10 and 50 ng/mL of DEX diminished these levels (*P*<0.05), suggesting that DEX can reduce MTX-induced excessive oxidative stress and iron overload in HT22 cells.

### DEX modulated the MTX-stimulated expression of ferritinophagy-related proteins in HT22 cells

**Figure 3:**
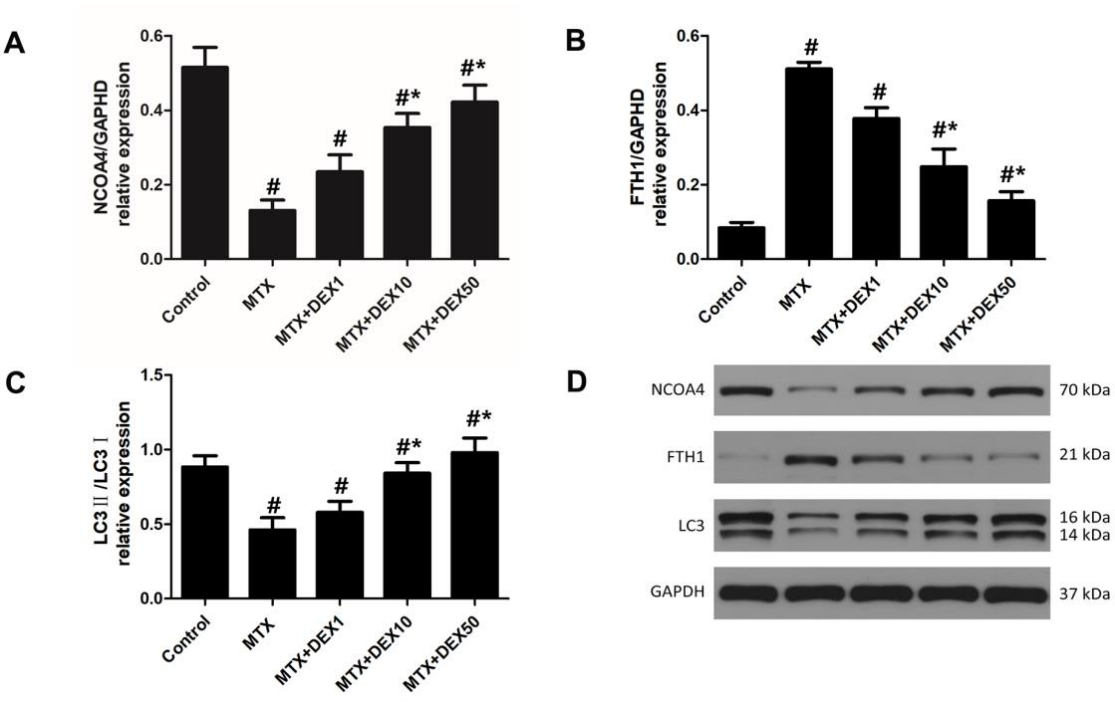
**DEX modulated the expression of ferritinophagy related protein in HT22 cells with MTX stimulation**. (**A**) Quantitative results of NCOA4, FTH1 (**B**) and LC3II/I (**C**) expression in HT22 cells with different treatments. n=3; #p<0.05, vs Control; *p<0.05, vs MTX group. (**D**) Western blot assay and representative results of NCOA4, FTH1 and LC3II/I in HT22 cells with different treatments.

In experiment 1, as depicted in Figure 3, we observed the expression of vital ferritinophagy-related proteins, including NCOA4, LC3-II, and FTH1. Accordingly, MTX markedly reduced the expression of NCOA4 and LC3-II (*P*<0.05) but triggered the accumulation of FTH1 in HT22 cells (*P*<0.05). Increasing DEX concentrations led to more significant decreases in the expression of FTH1 (*P*<0.05). Vital ferritinophagy markers, NCOA4 and LC3-II, were also up-regulated by DEX treatment (*P*<0.05), suggesting that ferritinophagy-related proteins may be involved in the protective effects of DEX on HT22 cells.

### DEX alleviated MTX-induced cytotoxicity and inflammatory injuries in HT22 cells via NCOA4-mediated ferritinophagy

**Figure 4:**
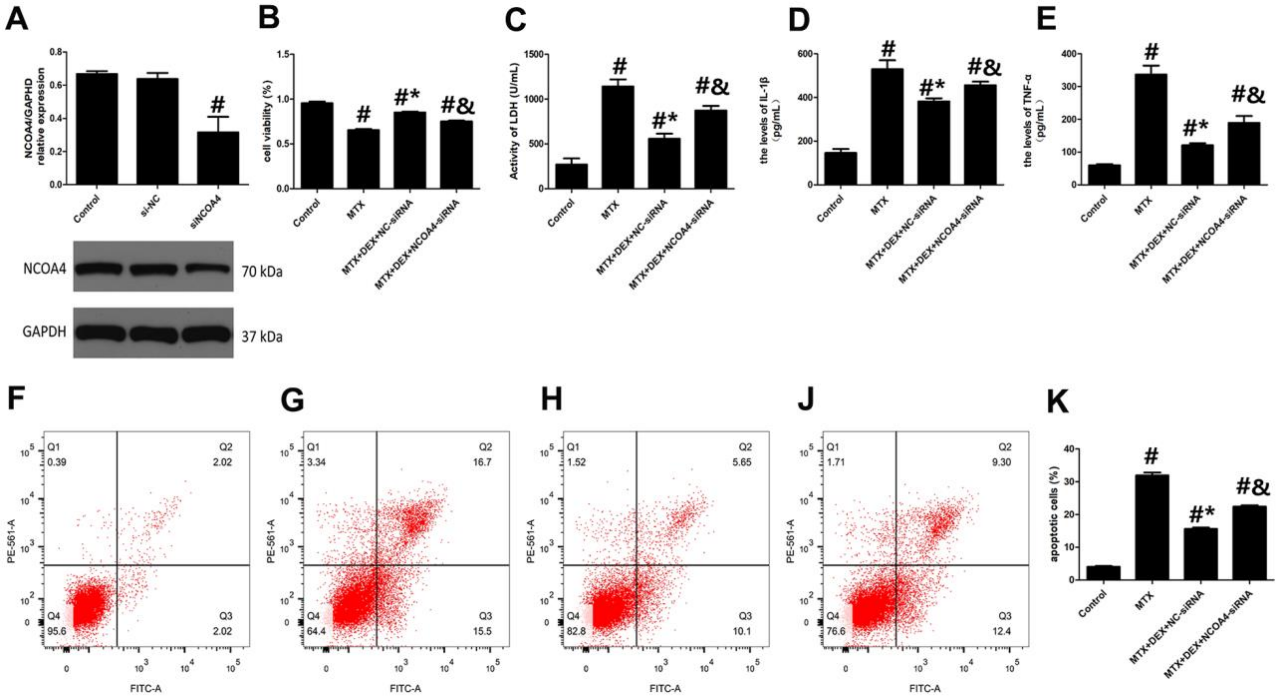
**DEX alleviated MTX-induced cytotoxicity and inflammatory injury in HT22 cells via NCOA4 mediated ferritinophagy.** (**A**) Representative bands of Western blot analysis for the expression of NCOA4 in HT22 cells treated with NCOA4-siRNA. (**B**) The cell viability of HT22 cells in MTX, DEX and NCOA4-siRNA group were measured by CCK-8 assay. (**C**) The activity of LDH in HT22 cells with MTX, DEX and NCOA4-siRNA were measured using LDH assay kit. (**D**) The levels of IL-1β and (**E**) TNF-α in HT22 cells with MTX, DEX and NCOA4-siRNA were measured using ELISA. (**F**) The representative results of apoptotic cells in control, MTX (**G**), MTX+50ng/mLDEX+NC-siRNA (**H**), and MTX+50ng/mLDEX+NCOA4-siRNA (**J**) group were tested by flow cytometry. (**K**) Flow cytometry analysis of HT22 cells in MTX, DEX and NCOA4-siRNA group. n=3; #p<0.05, vs Control; *p<0.05, vs MTX group; &p<0.05, vs MTX+DEX+NC-siRNA.

To further analyze the role of NCOA4-mediated ferritinophagy in DEX’s protection of HT22 cells, we silenced the expression of NCOA4 in HT22 cells with NCOA4 siRNA. NCOA4 siRNA and its negative control (NC) were transfected into the test and control HT22 cells, respectively. To ascertain transfection efficiency, western blot was utilized to confirm the effects of siRNA transfection. As described in Figure 4A, the expression of NCOA4 was significantly down-regulated in the NCOA4 siRNA group compared to the NCOA4 siRNA-NC group (*P*<0.05). Compared to the MTX+DEX group, the viability of HT22 cells in the NCOA4 siRNA group was considerably inhibited by NCOA4 siRNA (*P*<0.05, Figure 4B). Additionally, the protective effects of DEX on MTX-induced cytotoxicity in HT22 cells were also reversed by NCOA4 siRNA (*P*<0.05, Figure 4C). LDH release and apoptotic cells also increased in the NCOA4 siRNA group compared to the DEX+MTX group (*P*<0.05). Data from the above experiments show that NCOA4 siRNA abolished the protection of DEX on MTX-induced cytotoxicity in HT22 cells. Moreover, as illustrated in Figure 4D, 4E, NCOA4 siRNA boosted TNF-α and IL-1β levels in MTX- and DEX-co-treated cells (*P*<0.05). Data from these experiments suggest that NCOA4 siRNA can blunt DEX’s reversal of MTX-induced inflammation in HT22 cells.

**Figure 5:**
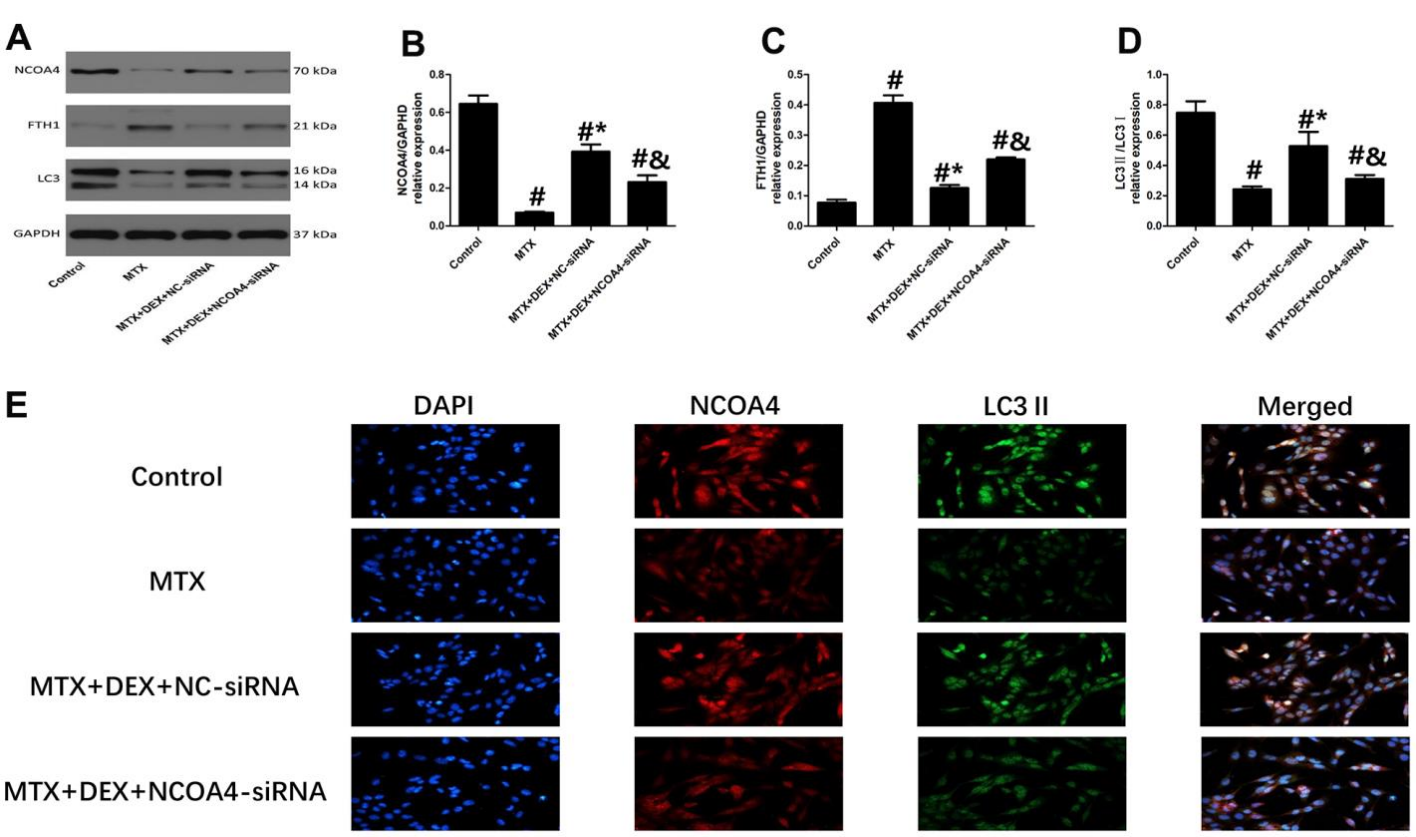
**Impact of NCOA4-siRNA on the expression of ferritinophagy related protein in HT22 cells with different treatments**. (**A**–**D**) Western blot assay and representative results of NCOA4, FTH1 and LC3II/I in HT22 cells with different treatments. n=3; #p<0.05, vs Control; *p<0.05, vs MTX group; &p<0.05, vs MTX+DEX+NC-siRNA (**E**) HT22 cells were co-stained with NCOA4 and LC3II. In representative images, NCOA4 and LC3II are shown in red and green, respectively.

Western blot and Immunofluorescence revealed that 50 ng/mL of DEX elevated the expression of ferritinophagy receptor-NCOA4 and LC3II in HT22 cells compared to cells treated with MTX (*P*<0.05, Figure 5B, 5E). By contrast, ferritinophagy substrate-FTH1 was substantially reduced by 50 ng/mL of DEX (*P*<0.05). However, the transfection of NCOA4-siRNA significantly decreased the expression of LC3 II and abolished the DEX-induced NCOA4 expression in HT22 cells (*P*<0.05, Figure 5A–5D). At the same time, the levels of FTH1 were up-regulated by NCOA4 siRNA treatment, revealing that the inhibition of NCOA4-mediated ferritinophagy eliminated DEX’s protection of MTX-stimulated HT22 cells.

### DEX alleviated MTX-induced iron aggregation and ROS overproduction in HT22 cells via NCOA4-mediated ferritinophagy

**Figure 6:**
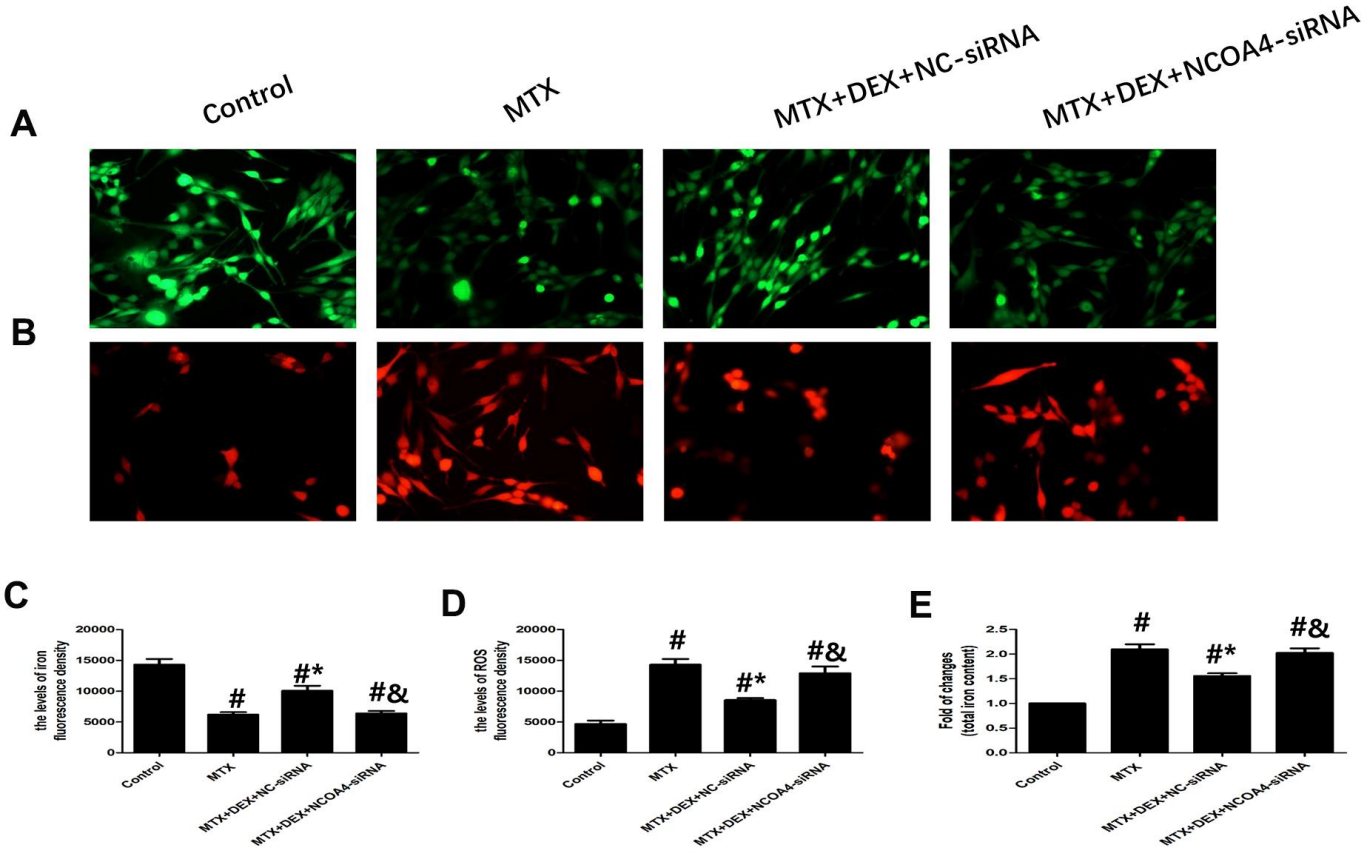
**DEX alleviated MTX-induced iron overload and ROS overproduction in HT22 cells via NCOA4 mediated ferritinophagy**. (**A**, **C**) Fluorescence microscope with Ca-AM probe analysis and quantitative results of iron fluorescence intensity in HT22 cells with different treatments. n=3; #p<0.05, vs Control; *p<0.05, vs MTX group; &p<0.05, vs MTX+DEX+NC-siRNA. (**B**, **D**) Fluorescence microscope with ROS probe analysis and quantitative results of ROS fluorescence intensity in HT22 cells with different treatments. n=3; #p<0.05, vs Control; *p<0.05, vs MTX group; &p<0.05, vs MTX+DEX+NC-siRNA. (**E**) Quantitative results of total iron content in HT22 cells with different treatments. n=3; #p<0.05, vs Control; *p<0.05, vs MTX group; &p<0.05, vs MTX+DEX+NC-siRNA.

Using NCOA4 siRNA, we examined whether NCOA4 is involved in MTX-induced iron overload and ROS overproduction in HT22 cells. As shown in Figure 6, introducing NCOA4 siRNA to HT22 cells stimulated by MTX and treated with DEX led to a notable rise in iron aggregation, including increased labile iron pool and total iron content (*P*<0.05), similar to observations in ROS overproduction in HT22 cells. Blunting the expression of NCOA4 saw ROS overproduced in HT22 cells stimulated by MTX and treated with DEX (*P*<0.05). These results suggest that the expression of NCOA4 is essential for DEX’s protection of HT22 cells.

## DISCUSSION

This research reveals that 10 and 50 ng/mL of DEX alleviated MTX-induced cytotoxicity and hippocampal neuronal inflammatory injuries. Also, DEX lightened MTX-induced iron overload and ROS overproduction in HT22 cells, pointing to its neuroprotective effects against MTX. Furthermore, the protective effects of DEX on MTX-induced cytotoxicity and inflammatory injuries in HT22 cells may be involved in NCOA4-mediated ferritinophagy. NCOA4 siRNA inhibited the neuroprotective property of DEX and disrupted iron homeostasis.

Excessive oxidative stress and neuroinflammation are thought to be involved in the pathogenesis of CICI, particularly after treatment with MTX. According to findings from a prospective cohort study, patients with acute lymphoblastic leukemia who received high-dose intravenous methotrexate sodium often had neuronal injuries [12]. The levels of brain injury biomarkers are potentially associated with worse neurologic outcomes, particularly those with genetic susceptibility to poor brain function. Reportedly, long-term treatment of rats with MTX instigates delayed deficits in their performance in Morris Water Maze and Novel Object Recognition tasks [13]. For juvenile animals, treatment with MTX supposedly leads to persistent deficits in spatial pattern memory, visual recognition memory, and executive function [14], with the potential mechanism possibly linked to neuroinflammation and microglial activation. Recent studies on MTX-induced neuroinflammation have mostly focused on the activation of microglia, astrocytes, and myelination. Allegedly, MTX disrupts oligodendrocyte lineage (OL) cell dynamics, myelin, and cognition in mice [15]. Moreover, according to the same research, MTX can induce chronic microglial activation and astrocyte reactivity, and inhibiting activated microglia can reverse glial cell dysregulation and cognitive deficits. The investigation further reveals that treatment with MTX causes a microglia-dependent reduction in Bdnf-TrkB expression, which is required for activity-regulated myelination. Using TrkB agonism could rescue cognitive performance after treatment with MTX [9]. In this study, through the evaluation of cell viability and the levels of pro-inflammatory cytokines, we confirmed that MTX induces cytotoxicity in HT22 cells and causes inflammatory injuries to them. Unlike other studies, we explored the mechanism of MTX-induced neurotoxicity and neuroinflammation from the perspective of ferritinophagy and iron homeostasis. Hopefully, this angle of our investigation benefits the exploration of potential therapeutic targets of CICI.

NCOA4-mediated ferritinophagy has gained added attention recently and is widely involved in various physiological processes associated with iron homeostasis. Disrupting the function of ferritinophagy causes a defect in iron metabolism that may contribute to excessive oxidative stress, leading to the occurrence of diseases, such as cardio-cerebrovascular diseases, tumors, and neurodegenerative diseases. In senescent cells, impaired ferritinophagy could promote iron accumulation and boost ferritin levels [16]. In chronic obstructive pulmonary disease pathogenesis, NCOA4-mediated ferritinophagy is initiated during ferritin degradation in response to cigarette smoke-induced epithelial cell ferroptosis [17]. In *in vivo* mouse models of NCOA4 depletion, iron accumulation in the liver and spleen accompanies increased levels of transferrin saturation, liver hepcidin, and serum ferritin [18]. In reality, iron contents in cells occur in different forms, including labile iron pool, ferritin, iron in phagosomes, etc.. In our study, we elected to measure only the labile iron pool, ferritin, and the total iron content (colorimetric determination) in our determination of the iron content in cells with different treatments, with the results potentially partially helpful with the issue of iron metabolism. Consistent with our findings, ferritin and the labile iron pool have been reported to be elevated in many pathological conditions, such as diabetic encephalopathy and lipopolysaccharide-induced inflammation in microglia [19, 20].

However, the expression and function of NCOA4 in the central nervous system and its role in neurodegenerative diseases is an unexplored area of research. Although there are few studies on NCOA4 expression levels or function during aging or in pathological specimens from patients with neurodegenerative diseases, many researchers believe that there is a potential link between NCOA4-mediated ferritinophagy and neurodegenerative diseases or neuroinflammatory diseases. In animal experiments, the likely cause of PD-associated retinal degeneration has been reported [7]. The over-expression of α-synuclein impairs ferritinophagy in the retina. Thus, iron overload is likely to initiate the spread of α-synuclein and ferritin, creating retinal iron dyshomeostasis and associated cytotoxicity. Therefore, inhibiting impaired ferritinophagy is a potential therapeutic approach in the treatment of neurodegenerative diseases or neuroinflammatory diseases.

In this investigation, we first presented the neuroprotective impact of DEX and the key role of NCOA4-mediated ferritinophagy in MTX-induced neurotoxicity and neuroinflammation. We observed that MTX disrupted NCOA4-mediated ferritinophagy, causing a decrease in the levels of NCOA4 and LC3-II and an accumulation of FTH1 in cultured HT22 cells. Although DEX protected against the MTX-induced impairment of ferritinophagy to some extent, NCOA4 siRNA reversed the protective effects of DEX on ferritinophagy and iron homeostasis.

Ferritin regulates the storage of iron and maintains the homeostasis of iron in cells, including FTL1 (ferritin light polypeptide 1) and FTH1. Current research generally suggests that FTH1 plays a major role in the capture of iron ions. Given the importance of the link between NCOA4 and FTH1 in intracellular iron homeostasis, recent studies have addressed the role of NCOA4 in physiological cellular processes—such as ferroptosis and erythropoiesis—that depend on iron availability [21, 22]. Ferroptosis is an iron-dependent form of regulated cell death and has recently been strongly linked to neurodegenerative diseases. Based on the link between NCOA4 and FTH1 in regulating intracellular iron levels, ferroptosis is thought to be modulated by NCOA4-ferritinophagy. We intend to examine the expression of ferroptosis-related biomarkers next to provide a reference for the pathogenesis of MTX-induced neuronal injuries and neurodegenerative diseases.

Due to its favorable physiological properties and limited adverse effects, DEX has gradually become an attractive adjunct to clinical anesthesia and an important option for sedation in intensive care settings. For sepsis, many animal investigations have shown that DEX decreases mortality and inhibits inflammation by modulating the activity of the immune system and lowering cytokine concentrations. Moreover, DEX appears to have a multiple organ protective role for the heart, brain, and kidney, as well as a beneficial role for the functioning of the intestine and liver, as it lessens systemic inflammation and increases the survival rate in septic rats [23–25]. Regarding postoperative cognitive dysfunction (POCD) and postoperative delirium (POD), there is proof of favorable outcomes using DEX over placebo for the reduction of POD [26]. Animal models have revealed that DEX’s potential neuroprotective mechanism against POD may be involved in anti-inflammatory and immunomodulatory effects. Although intraoperative DEX infusion does not improve renal function in terms of serum Cr-related indices following cytoreductive surgery and hyperthermic intraperitoneal chemotherapy, DEX can attenuate decreased creatinine clearance and lower early tubular-injury markers, which may be a protective property against tubular injuries in cytoreductive surgery and hyperthermic intraperitoneal chemotherapy [27]. Our current research provides some pieces of evidence that DEX has a protective role when combined with chemotherapy drugs. Despite the current lack of clinical assessments of the impact of DEX on CICI, findings from animal investigations suggest that DEX can subdue CICI by modulating miR-429-3p and mitochondrial DNA gene mt-ND1 expression and decreasing caspase-9 expression in rats [28]. These animal studies provide references for the selection of adjuvant chemotherapy drugs and the treatment of CICI.

There are some limitations to the present study. First, we did not investigate the downstream regulatory mechanism of NCOA4-ferritinophagy in CICI. Perhaps the anti-oxidative and anti-inflammatory effects of NCOA4-ferritinophagy are linked to its ferroptosis activity and relative stimulation of some RNA-binding proteins. Furthermore, animal studies are needed to elucidate the exact mechanism of CICI. Second, we found that post treatment with DEX remarkably inhibited MTX-triggered excessive oxidative stress and neuroinflammation in HT22 cells. However, we also observed that pretreatment with DEX further attenuates lipopolysaccharide-induced pro-inflammatory response in primary microglia. Therefore, it is necessary to further investigate the differences in the anti-inflammatory properties of DEX between pretreatment and post treatment.

## CONCLUSIONS

DEX weakened MTX-provoked neurotoxicity and neuroinflammatory injuries in HT22 cells, possibly by modulating NCOA4-mediated ferritinophagy. Our discoveries present further mechanisms for understanding the protective effect of DEX against MTX-induced cognitive impairment.

## MATERIALS AND METHODS

### Hippocampal neuronal cell culture, drug treatment, and transfection

Mouse hippocampal HT22 cell lines were purchased from Wuhan Hycell biotech and cultured in Dulbecco's modified Eagle's medium (DMEM) (HyClone, UT, USA) with 1% penicillin/streptomycin (HyClone, UT, USA) and 10% fetal bovine serum (HyClone, UT, USA) in an incubator (Herocell 180, RADOBIO, Shanghai, China) at 37° C and 5% CO_2_. The cultured HT22 cells were then seeded in 96-well plates at a density of 1*10^5^ cells/mL, stimulated with MTX (Pfizer, NY, USA), and treated with different concentrations of DEX (Hengrui, Jiangsu, China), depending on experimental conditions. Based on literature reports on the use of MTX [29] in clinical therapeutics and experiments, including *in vivo* and *in vitro* tests, we used 100 μM MTX to stimulate HT22 cells for 24 h to create the neurotoxicity and inflammation model. In the first experiment, we utilized various concentrations (1, 10, or 50 ng/mL) of DEX for 1 h after 24 h of MTX stimulation. In the second experiment, HT22 cells first received small interfering RNA (siRNA) transfection. HT22 cells were transfected with NCOA4-siRNA or non-targeting siRNA (RIBOBIO, Guangzhou, China) for 48 h using Lipofectamine 3000 reagent (Invitrogen, CA, USA). The sequences of the siRNAs used were as follows: siRNA-NCOA4 5'-GCTGGGAAACCAGCGAGAAGTTTAA-3'; siRNA-NC 5'-TACGGCTCGAAGGACAACTATTTAA-3'. Following transfection, HT22 cells were stimulated with MTX for 24 h and then treated with 50 ng/mL DEX for 1 h.

### HT22 cell viability assay

We assessed HT22 cell viability using the CCK-8 kit (Dojindo, MD, USA) according to the manufacturer’s instructions. Tetrazolium cell viability assays rely on cellular dehydrogenases to form a colored formazan product, which is measured by absorbance. After treatment with different drug concentrations, 10 μL of the CCK-8 solution was added to each well of the plate in an incubator for 60 min at 37° C and 5% CO_2_. Absorbance was measured at 450 nm using a microplate reader (Model 550; Bio-Rad). This assay was carried out three times.

### LDH assay

We evaluated the level of lactate dehydrogenase (LDH) using the LDH assay kit (Beyotime, Shanghai, China) according to the manufacturer’s protocol. After treatment with different concentrations of DEX for 1h, 50 μL of the supernatant from each well was moved to a new 96-well plate. The LDH-assay reaction solution was then added to the plate. Absorbance was measured at 490 nm using a microplate reader. This assay was carried out three times.

### Cell apoptosis assay

We determined HT22 cell apoptosis using the Annexin V-FITC Apoptosis Kit (BD Biosciences, CA, USA) and a CELLQuest software from FACSCalibur (BD Biosciences, CA, USA). Per instructions from the supplier’s protocol, we harvested the cells and removed the supernatant from each well. Subsequently, we suspended the cells in PBS and centrifuged for 5 min at 2200 rpm. After centrifugation, we suspended the cells in 300 μL of binding buffer and incubated with 50 μL Annexin V-FITC and 5 μL PI in the dark at room temperature for 10 min and 5 min, respectively. The apoptotic index was analyzed with FACSCalibur within 1 h. This assay was carried out three times.

### ELISA

We assessed the levels of tumor necrosis factor-α (TNF-α), and interleukin-1β (IL-1β) in the supernatants of cultured cells using the relevant ELISA kit (R&D Systems, MN, USA) according to the manufacturer’s instructions. Absorbance was measured at 450 nm using a microplate reader. This assay was carried out three times.

### Western blot

To determine protein concentration, HT22 cells were first lysed in a lysis buffer using a BCA kit (Pierce, MA, USA) according to standard protocols. Protein samples were severed and separated with 10% SDS-PAGE and transferred onto PVDF membranes where they were blocked with 5% commercial skim milk at room temperature for 1 h and immunoblotted with primary antibodies for NCOA4 (1:500, SANTA CRUZ, sc-373739), Ferritin heavy chain 1 (FTH1) (1:1000, Abcam, ab65080), LC3 (1:1000, CST, #4108), and GAPDH (1:10000, Abcam, ab37168) overnight at 4° C. The following morning, the membranes were washed and incubated with HRP secondary antibodies (1:10000, ASPEN, AS1107) at room temperature for 1 h. Protein concentration was detected using the Molecular Imager VersaDoc MP 5000 System (Bio-Rad Life Science Research, Hercules, CA) and analyzed with ImageJ software (Version 1.50i, USA).

### ROS assay

HT22 cells from different groups were cultivated in a 6-well plate. Following instructions from the ROS kit protocol (Beyotime, China), 1 mL of 2',7'-Dichlorodihydrofluorescein diacetate (DCFH-DA) (10 mM) was added to the plate and incubated in the dark at 37° C for 20 min. The resulting supernatants were discarded, and ROS was determined using flow cytometry (Guava Technologies, Inc., Millipore, USA).

### Total iron content determination in HT22 cells

We determined the content of iron in cells by colorimetry using an iron assay kit from Nanjing Jiancheng according to the instructions from the manufacturer’s protocol. Free iron ions in cell lysates combined with dipyridine, forming a pink complex. Because the maximum absorption peak was 520 nm, the content of free iron ions in HT22 cells was determined using the final absorbance OD value obtained at 520 nm.

### Intracellular labile iron pool determination

Intracellular labile iron pool determination was performed using a Calcein-acetoxymethyl ester (Ca-AM) probe (Beyotime, China), a green fluorescent indicator. Treated-HT22 hippocampal cells were centrifuged at 250 g at room temperature for 5 min, and the resulting supernatant was discarded. The 1 μM Calcein AM probe was then added to the cells and incubated at 37° C for 40 min. After incubation, the cells were centrifuged twice, each time at 250 g at room temperature for 5 min. Intracellular iron was observed and adjudged with a fluorescence microplate reader microscope (BMG, Germany) based on the green fluorescence intensity (494/514 nm). The level of the labile iron pool is inversely proportional to fluorescence intensity.

### Immunofluorescence co-localization analysis

HT22 cells were fixed with 4% paraformaldehyde at room temperature for 30 min, blocked with 1% BSA, and then immunostained with the following primary antibodies: NCOA4 (1:500), FTH1 (1:1000), and LC3 II (1:1000) and secondary antibodies (mouse and rabbit) (1:1000). Fluorescent images were taken with a fluorescence microscope (OLYMPUS, Japan). This assay was carried out three times. Representative images are shown in the Figures.

### Statistical analysis

All results are presented as the mean ± SD. One-way ANOVA was used for comparisons between groups, and the Bonferroni test was used for multiple comparisons between groups. Differences were considered statistically significant when *P*<0.05. The SPSS 17.0 statistical software (SPSS, Inc., Chicago, IL, USA) was utilized to analyze results.

### Data availability

The datasets analyzed during the current study are available from the corresponding author on reasonable request.
